# Soluble urokinase plasminogen activator receptor for risk stratification from undifferentiated acute chest pain through to convalescence after acute coronary syndromes

**DOI:** 10.1093/ehjopen/oeaf097

**Published:** 2025-08-07

**Authors:** Taylor Keys, Charlotte Greer, Philip D Adamson, John W Pickering, Anna P Pilbrow, Christopher Frampton, Salim Hayek, Richard W Troughton, Robert N Doughty, A Mark Richards, Christopher J Pemberton, Janice Chew-Harris

**Affiliations:** Department of Medicine, Christchurch Heart Institute, University of Otago, 2 Riccarton Avenue, Christchurch 8011, New Zealand; Department of Medicine, Christchurch Heart Institute, University of Otago, 2 Riccarton Avenue, Christchurch 8011, New Zealand; Department of Medicine, Christchurch Heart Institute, University of Otago, 2 Riccarton Avenue, Christchurch 8011, New Zealand; British Heart Foundation Centre for Cardiovascular Science, University of Edinburgh, 47 Little France Crescent, Edinburgh EH16 4TJ, UK; Department of Medicine, Christchurch Heart Institute, University of Otago, 2 Riccarton Avenue, Christchurch 8011, New Zealand; Department of Medicine, Christchurch Heart Institute, University of Otago, 2 Riccarton Avenue, Christchurch 8011, New Zealand; Department of Medicine, Christchurch Heart Institute, University of Otago, 2 Riccarton Avenue, Christchurch 8011, New Zealand; Department of Internal Medicine, University of Texas Medical Branch, 301 University Blvd, Galveston, TX 77555, USA; Department of Medicine, Christchurch Heart Institute, University of Otago, 2 Riccarton Avenue, Christchurch 8011, New Zealand; Greenlane Cardiovascular Service, Te Toka Tumai Auckland Hospital, 2 Park Road, Grafton, Auckland 1023, New Zealand; Heart Health Research Group, Department of Medicine, University of Auckland, 85 Park Road, Grafton, Auckland 1023, New Zealand; Department of Medicine, Christchurch Heart Institute, University of Otago, 2 Riccarton Avenue, Christchurch 8011, New Zealand; Department of Medicine, Christchurch Heart Institute, University of Otago, 2 Riccarton Avenue, Christchurch 8011, New Zealand; Department of Medicine, Christchurch Heart Institute, University of Otago, 2 Riccarton Avenue, Christchurch 8011, New Zealand

**Keywords:** suPAR, Concentrations, ACS, Risk, Cardiac biomarkers

## Abstract

**Aims:**

The performance of plasma soluble urokinase plasminogen activator receptor (suPAR) for prediction of heart failure (HF) readmission or death within 5 years was assessed in patients incurring (i) initially undifferentiated chest pain and (ii) immediately after diagnosed with acute coronary syndromes (ACS), and (iii) in recovery after ACS.

**Methods and results:**

Soluble urokinase plasminogen activator receptor concentrations were measured at admission for acute chest pain patients (*n* = 917) including confirmed ACS (26.5%) and in an independent 4–6-week post-ACS cohort (*n* = 1301). Soluble urokinase plasminogen activator receptor’s prognostic performance, in comparison, and combination with cardiac troponin I or *n*-terminal-pro B-type natriuretic peptide (NT-proBNP) was evaluated by risk discrimination, hazard ratios (HRs) as continuous variables, and cut-off concentrations across the three settings in unadjusted and adjusted analyses. In acute undifferentiated chest pain including the subgroup with confirmed ACS and separately post-ACS convalescence, combining suPAR and NT-proBNP yielded the highest discriminatory power for endpoints (c-statistics >0.80, ≥Δ0.02). Soluble urokinase plasminogen activator receptor in the acute and post-ACS convalescent conferred additional hazard for the composite endpoint of HF/death (HR > 1.4), HF (HR > 1.3), and death (HR > 1.2) after adjustment for risk factors including circulating cardiac markers. Although suPAR > 3.65 ng/mL (>83% specificity, > 91% negative predictive value (NPV) was superior in admission-ACS (HR, 10.5) for risk of HF/death, independent prediction for the composite endpoint remained consistent across the three settings and in men.

**Conclusion:**

Plasma suPAR independently predicted risk of readmissions with HF and death, independent of key clinical indicators and cardiac markers at all points of the patient journey from acute chest pain presentation through to post-ACS convalescence. Its use in acute chest pain and ACS may augment risk stratification strategies.

## Graphical Abstract

**Graphical Abstract:**
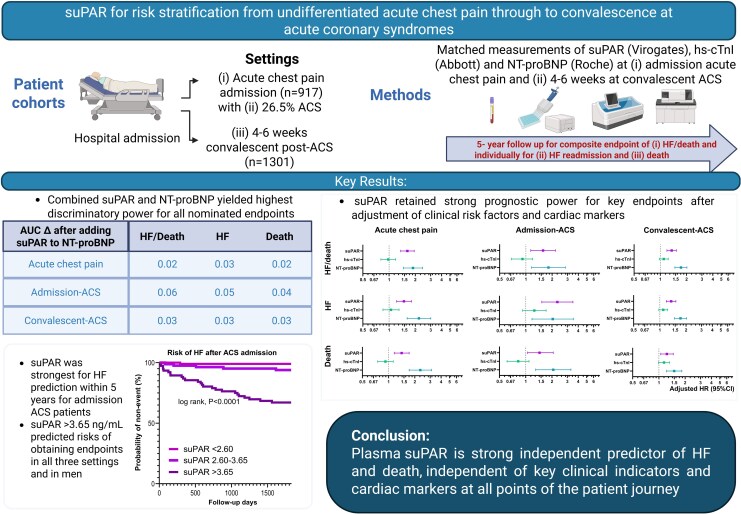

## Introduction

Acute chest pain is a common presentation to the emergency department. A serious underlying cause for this symptom is acute coronary syndrome (ACS), diagnosed in ∼15–25% of cases. Continuing advances in rapid diagnosis, early revascularization, deployment of effective adjunct pharmacotherapies, and post-discharge prevention have led to improved ACS outcomes; however, many survivors remain at significant risk of long-term morbidity and mortality.^1,2^ For ACS patients, despite effectiveness of emergent revascularization in immediate restoration of myocardial perfusion,^3^ progressive atheromatous disease poses ongoing residual risks of recurrent ACS events with ≥50% of new acute occlusions occurring in untreated areas of the coronary vasculature. Additionally, heart failure (HF) remains a common severe consequence of ACS, occurring in over one-third of acute myocardial infarction (MI) patients.^4^ As coronary artery disease (CAD), primarily due to atherosclerosis (a multi-faceted process with multiple drivers) is the precursor to ACS^5,6^ accounting for multiple factors involved in disease processes may enhance risk stratification and, consequently, direct management to improve patient outcomes.

Soluble urokinase plasminogen activator receptor (suPAR) is an integral component in the modulation of fibrinolysis.^7,8^ Increments in circulating suPAR concentrations reflect activation of various immunologically active cells and have been implicated in the formation of atherosclerotic lesions and plaque destabilization.^9–12^ In healthy individuals, plasma suPAR independently predicts future cardiovascular disease and all-cause death.^13^ Limited evidence suggests that plasma suPAR concentrations are associated with subsequent ACS events and all-cause death in patients undergoing angiography for suspected CAD.^14^ Thus, suPAR is a candidate marker for improving risk stratification in acute chest pain and post-ACS. However, the majority of studies evaluating suPAR in risk prediction have assessed plasma concentrations measured during hospital admission.^15,16^ As acute illnesses may cause initial volatile perturbations in markers,^17^ measuring suPAR in the post-acute phase should also be examined to fully evaluate its performance as a marker of longer-term risk.

This study undertook a multi-staged approach incorporating sampling for suPAR at key time points in the clinical journey, aimed at delineating suPAR’s ability to improve risk stratification from the acute phase setting through to post-ACS convalescence. We determined suPAR’s prognostic performance when sampled at acute chest pain hospital admission including those adjudicated with ACS, and separately at 4–6 weeks of recovery in an independent post-ACS cohort. We compared the prognostic performance of suPAR for 5-year endpoints of HF readmission and all-cause death to that of established cardiac markers cardiac troponin I (cTnI) and *n*-terminal-pro B-type natriuretic peptide (NT-proBNP). Finally, a selected threshold concentration of suPAR was assessed for generalized application across the three settings for predicting nominated endpoints.

## Methods

### Study cohorts

#### Undifferentiated acute chest pain including confirmed acute coronary syndrome

A total of 917 eligible undifferentiated acute chest pain patients, aged ≥18 years, admitted to Christchurch Hospital for at least 24 h, clinically suspicious of ACS and ≤4 h from onset, were recruited in our ongoing observational study called Signal Peptide in Acute Coronary Events (ACTRN12609000057280),^18^ between November 2007 and September 2018. Undifferentiated chest pain was defined as chest pain prior to definitive diagnosis. All patients gave informed consent and were enrolled according to protocols approved by the Health and Disabilities Ethics Committee of the Ministry of Health, New Zealand (NZ).

Acute MI diagnosis was made in accordance with published guidelines and cTnI data by two independent cardiologists.^3^ A third independent cardiologist adjudicated any disagreement. The diagnosis of unstable angina (UA) was based on confirmatory provocative investigations (exercise tolerance testing or dobutamine stress echocardiography) or angiographic catheterization findings.^19^ The terminology ‘admission-ACS’ was used to describe patients with confirmed ACS from this acute chest pain setting. Presentations comprising conduction disorders (sick sinus syndrome), arrhythmias, and acute HF were classified into ‘other cardiac disorders’. Non-cardiac chest pain was defined as present when a definite non-cardiac cause for symptoms was identified.^18^

#### Post-acute coronary syndrome convalescence cohort

Plasma samples from a total of 1301 patients presenting with ACS recruited into the Coronary Disease Cohort Study (CDCS) in Christchurch and Auckland City Hospitals (NZ) between 2002 and 2009, were used for convalescent suPAR measurements. The CDCS was designed to explore determinants of long-term cardiovascular risk and establish—relationships between convalescent circulating biomarkers with clinical outcomes, especially HF, and all-cause death (ACTRN12605000431628). Inclusion criteria comprised both ischaemic discomfort and one or more ECG changes, elevated cardiac marker levels, history (hx) of coronary disease, or ≥65 years of age in patients with diabetes or vascular disease.^20^

The CDCS included a broad spectrum of subgroups with important antecedent risk factors and disease processes such as hypertension and diabetes. Index hospital admission clinical data were available, with further clinical and neurohormonal data collected at ‘baseline’ median 29 days after ACS for the current analysis, a deliberate delay to specifically allow convalescent marker assessments. A ‘convalescent-ACS’ terminology was used to reflect the timing of biomarker measurements and clinical data collection.

### Plasma analytes

Following collection and centrifugation, separated venous EDTA plasma samples were stored at −80°C until analysis for all biomarkers. Before analyte measurements, all plasma samples were re-centrifuged. Soluble urokinase plasminogen activator receptor concentrations were measured using the 2-site suPARnostic® AUTO Flex enzyme-linked immunosorbent assay by ViroGates (Birkerød, Denmark) in aliquots with less than three freeze–thaw cycles, an upper limit not affecting measured concentrations.^21^ The assay’s detection limit is 0.1 ng/mL, and previous in-house assay performance analyses found coefficient of variation (CV) of 3.6% (inter-assay) at 3.9 ng/mL of suPAR.^21^ NT-proBNP concentrations were measured by an electrochemiluminescent assay using an Elecsys Cobas e411 (Roche) with an inter-assay CV < 2.0% at 88 pmol/L, whilst cTnI concentration was determined using the ARCHITECT STAT high-sensitivity (hs-cTnI) assay (Abbott Laboratories), with an interassay of CV < 10% at 1.2 ng/L.

### Statistical analysis

All long-term clinical events (HF hospitalizations and all-cause death) at follow-up were determined from the NZ National Health Information Services databases, comprising comprehensive population-wide records linked by unique identifiers. Results are reported for the three settings comprising (i) pre-diagnosis acute chest pain, (ii) the subset with confirmed admission-ACS, and (iii) the independent convalescent-ACS group.

Baseline patient characteristics for each setting are reported at the hospital admission time-point representing the acute phase for the chest pain cohort including the admission-ACS subcohort and separately, 4–6 weeks post-index admission representing concentrations for the convalescent-ACS setting. Values are expressed as means (standard deviations), medians (interquartile range (IQR)), or counts and percentages as appropriate. Skewed data were normalized by logarithmic transformation. Soluble urokinase plasminogen activator receptor’s correlation with clinical variables [Spearman’s rho (*r*_s_) and general linear model] was assessed for all settings.

Using a 95% central distribution of suPAR of 1.6–3.6 ng/mL, previously determined in our study of healthy individuals (*n* = 155) aged 17–70 years without any evidence of cardiovascular, endocrine, or psychiatric illness,^21^ any values of >3.65 ng/mL in the current study were classified as the upper range, 2.60–3.65 ng/mL as the mid-cut-off range, and <2.60 ng/mL as the low range. Sensitivity, specificity, positive predictive value and negative predictive value (NPV) were calculated for suPAR > 3.65 ng/mL. Kaplan–Meier event-free survival curve analyses were used to summarize clinical endpoints for suPAR ranges.

Risk discrimination (c-statistics) was used to compare suPAR, hs-cTnI, and NT-proBNP for all endpoints as individual markers and in combination in unadjusted analysis. Markers were combined additively by generating a probability score, whereby combinations of two biomarkers were forced into binary logistic regression for the associated outcome at 5 years, and differences in c-statistic between the combined markers were determined. Relationships between suPAR and cardiac biomarkers with clinical endpoints were assessed using Cox proportional hazard models, reported as hazard ratios (HRs) with 95% confidence intervals (CIs) expressed as per doubling in plasma concentration of each biomarker for all settings. When assessed together, concentrations of biomarkers were standardized to a Z-score where a difference of 1 refers to per doubling of the concentrations. Hazard ratios were also assessed according to suPAR and individual cardiac biomarker cut-offs, which used the lowest cut-off values of each as the referent or otherwise specified. Predetermined ranges were <5, 5–14.9, and ≥15 ng/L for hs-cTnI and <50, 50–99.9 and ≥100 pmol/L for NT-proBNP.

In multivariable analyses, models were adjusted for age, sex, heart rate, systolic blood pressure (SBP), hx-HF, hx-MI, diabetes mellitus, hypertension, in-hospital percutaneous coronary intervention (PCI), taking a second antiplatelet therapy, and plasma creatinine measured at the same time-point as biomarkers. Covariates for adjustment were chosen based on inclusion in the validated Global Registry of Acute Coronary Events score for survival prediction after index ACS. Creatinine concentrations were selected over estimated glomerular filtration rate (eGFR) to avoid over-fitting age in multivariable analyses. Complete datasets required for multivariable modelling were available for all patients. In a separate analysis, Fine and Gray Cox regression modelling was used to examine suPAR’s prognostic ability to predict HF as an outcome, adjusting for the abovementioned factors and accounting for all-cause death as a competing risk. For the admission-ACS subcohort where a limited number of clinical events may be present, a probability score was generated under binary logistic regression for the respective nominated endpoint by combining the abovementioned variables and adding biomarkers as appropriate. Under Cox regression for any nominated endpoint in admission-ACS, the independent biomarker was assessed as a separate variable in the presence of the derived probability score as the second variable. Results were also presented according to sex and those with renal insufficiency, defined as having eGFR < 60 mL/min/1.73m^2^, to determine suPAR’s upper threshold generalizability for predicting the composite outcome of HF/death.

Statistical analysis was conducted using IBM SPSS Statistics version 29 and R 4.3.3 (R Core Team, Vienna, Austria), with graphical presentations designed using GraphPad Prism 10.4.0.

## Results

*Table 1* details baseline characteristics for all three settings. For acute chest pain patients, a total of 179 patients were diagnosed with acute MI {ST-elevation myocardial infarction [STEMI] (*n* = 26), non-ST-elevation myocardial infarction [NSTEMI] (*n* = 153)}, and 64 patients with UA. Index presentations for the convalescent-ACS cohort comprised 312 (24.0%) STEMI, 634 (48.7%) with NSTEMI, and 355 (27.3%) with UA. Median age was 63 years (IQR: 54–73) for acute chest pain patients, 66 years (58–75) for admission-ACS, and 66 years (56–76) for the convalescent-ACS cohort.

**Table 1:** Clinical characteristics of the acute chest pain cohort, the admission-ACS subcohort and the convalescent post-ACS cohort

	Acute chest pain (*n* = 917)	Admission-ACS (*n* = 243)	Convalescent-ACS (*n* = 1301)
Age (years)	63 (54–74)	66 (58–75)	66 (56–76)
Male sex (%)	601 (65.5)	178 (73.3)	941 (72.3)
BMI (kg/m^2^)	28 (25–32)	28 (25–31)	27 (24–30)
suPAR (ng/mL)	2.7 (2.2–3.6)	3.0 (2.3–4.0)	3.1 (2.4–4.1)
NT-proBNP (pmol/L)	21 (7–60)	34 (13–105)	80 (37–162)
Troponin I (ng/L)	4.4 (2.0–15.6)	45.0 (10.7–170.7)	8.8 (4.6–20.0)
Heart rate (b.p.m.)	70 (62–81)	70 (62–80)	61 (55–69)
Systolic BP (mmHg)	141 (126–162)	145 (128–167)	126 (112–140)
Diastolic BP (mmHg)	79 (70–90)	80 (71–92)	72 (66–81)
Creatinine (µmol/L)	89 (80–100)	93 (82–106)	90 (80–110)
eGFR^a^ (mL/min/1.73 m^2^)	60 (51–70)	56 (48–66)	73 (62–83)
Killip class, *n* (%)			
Class I	848 (92.5)	222 (91.3)	988 (75.9)
Class II	44 (4.8)	14 (5.8)	292 (22.4)
Class III/IV	2 (0.2)	1 (0.4)	22 (1.7)
Unknown	23 (2.5)	6 (2.5)	—
**Index diagnosis, *n* (%)**			
STEMI	26 (2.8)	26 (10.7)	312 (24.0)
NSTEMI	153 (16.7)	153 (63.0)	634 (48.7)
UA	64 (7.0)	64 (26.3)	355 (27.3)
Other cardiac chest pain	49 (5.3)	-	-
Non-cardiac chest pain	625 (68.2)	—	—
**Medical history, *n* (%)**			
Diabetes	132 (14.4)	41 (16.9)	219 (16.8)
Hypertension	532 (58.0)	160 (65.8)	652 (50.1)
History of MI	274 (29.9)	94 (38.7)	393 (30.2)
History of HF	73 (8.0)	18 (7.4)	121 (9.3)
**Patient management, *n* (%)**			
PCI during admission	141 (15.4)	141 (58.0)	608 (46.7)
Second antiplatelet therapy	265 (28.9)	201 (82.7)	548 (42.1)

^a^eGFR was calculated from the CKD-EPI equation for the acute chest pain cohort and from the MDRD equation for the convalescent-acute coronary syndrome cohort. Variables presented correspond to the timing of suPAR sampling. Data is presented as median (interquartile range) or *n* (%). For those on second antiplatelet therapy in the acute chest pain and admission-acute coronary syndrome cohort, patients are either on a combination of aspirin and clopidogrel or a combination of aspirin and ticagrelor. For patients in the post-acute coronary syndrome cohort, those on second antiplatelet therapy received a combination of aspirin and clopidogrel.

eGFR, estimated glomerular filtration rate; CKD-EPI, chronic kidney disease epidemiology collaboration; MDRD, modification of diet in renal disease; ACS, acute coronary syndromes; BMI, body mass index; suPAR, soluble urokinase plasminogen activator receptor; NT-proBNP, *n*-terminal-pro B-type natriuretic peptide; BP, blood pressure; STEMI, ST-elevation myocardial infarction, NSTEMI, non-ST-elevation myocardial infarction; UA, unstable angina; HF, heart failure; PCI, percutaneous coronary intervention.

The median plasma concentration of suPAR for the overall acute chest pain cohort was 2.7 ng/mL (IQR: 2.2–3.6), 3.0 ng/mL (2.3–4.0) in admission-ACS, and 3.1 ng/mL (2.4–4.1) in convalescent-ACS. Higher suPAR concentrations were obtained in women [median: 3.0 ng/mL (2.4–3.8) in chest pain, 3.4 ng/mL (2.8–4.1) in admission-ACS, and 3.4 ng/mL (2.7–4.4) in convalescent-ACS) than in men (2.6 ng/mL (2.1–3.5) in chest pain, 2.7 ng/mL (2.2–3.7) in admission-ACS, and 2.9 ng/mL (2.3–3.9) in convalescent-ACS] (all *P* < 0.0001). Regardless of patient settings, higher suPAR associated with increasing age, diabetes, hypertension, hx-HF, hx-MI and lower eGFR.

Median NT-proBNP concentrations were 21 pmol/L (7–60) in acute chest pain, 34 pmol/L (13–105) in admission-ACS, and 80 pmol/L (37–162) in convalescent-ACS. Median hs-cTnI concentrations were 4.4 ng/L (2.0–15.6), 45 ng/L (10.7–170.7), and 8.8 ng/L (4.6–20.0) for acute chest pain, admission-ACS, and convalescent-ACS, respectively. NT-proBNP correlated with suPAR in acute chest pain [*r*_s_ = 0.510 (95% CI: 0.459–0.558)], admission-ACS [*r*_s_ = 0.450 (0.340–0.547)], and convalescent-ACS [*r*_s_ = 0.490 (0.446–0.531)]. Hs-cTnI also correlated with suPAR in acute chest pain [*r*_s_ = 0.303 (0.241–0.632)], admission-ACS [*r*_s_ = 0.151 (0.02–0.275)], and convalescent-ACS [*r*_s_ = 0.251 (0.197–0.302)]. Common independent determinants of plasma suPAR in all three defined groups were age, sex, and plasma creatinine (see Supplementary material online, *Table S1*).

### Soluble urokinase plasminogen activator receptor and clinical endpoints

**Figure 1:**
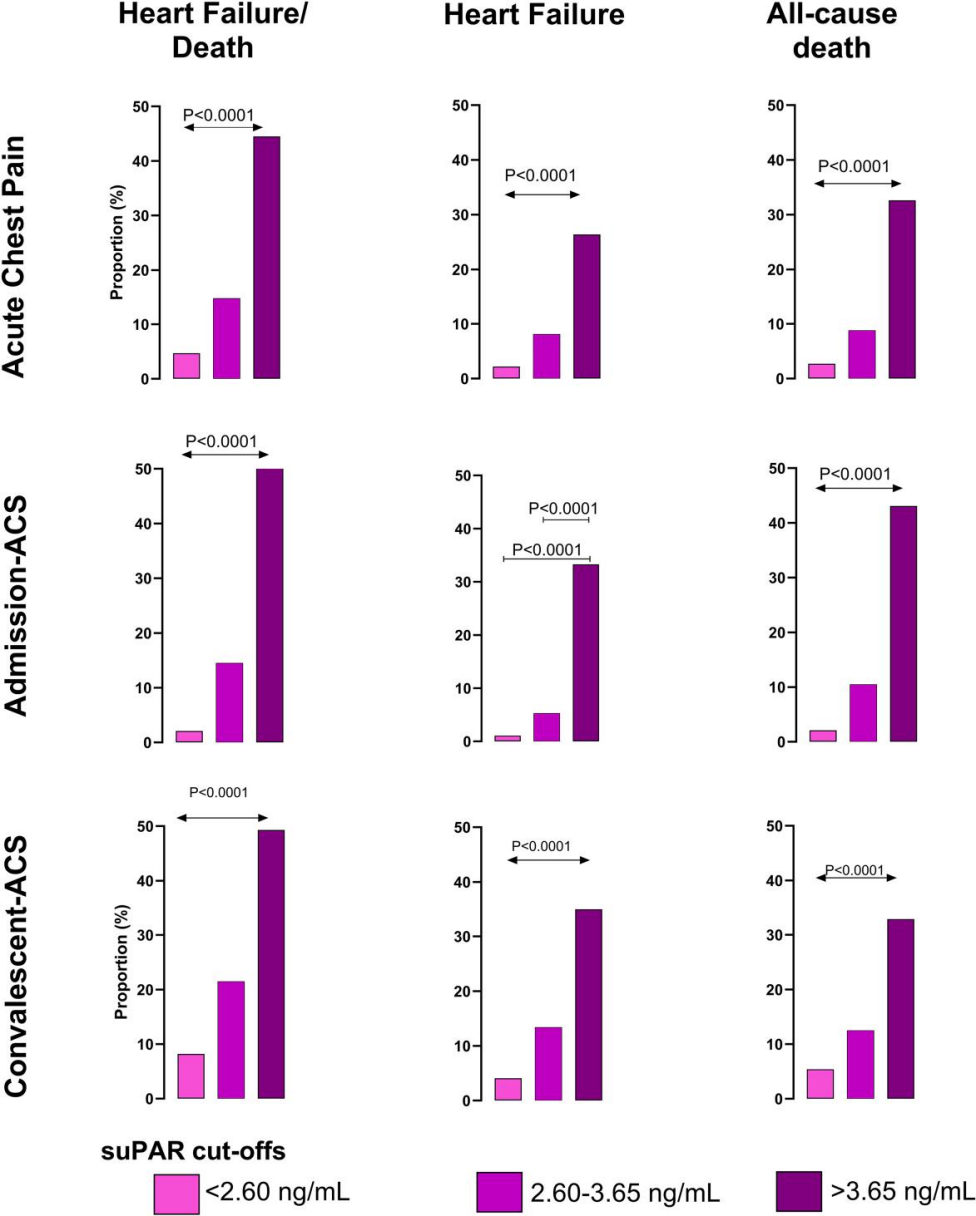
Bar graphs depict the proportion of patients incurring outcomes at 5 years according to suPAR cut-off levels for acute chest pain patients (*n* = 917), in the subcohort with admission-acute coronary syndrome (*n* = 243) and separately in the convalescent-acute coronary syndrome cohort (*n* = 1301). Ascending suPAR cut-off levels are associated with increased rates of the composite endpoint of heart failure/all-cause death, heart failure, and all-cause death. Double-headed arrow denotes differences across all levels, i.e. increasing rates of all outcomes for those between suPAR > 3.65 ng/mL and suPAR 2.6–3.65 ng/mL, between suPAR 2.60–3.65 ng/mL and suPAR < 2.6 ng/mL, and between suPAR > 3.65 ng/mL and suPARr < 2.6 ng/mL. ACS, acute coronary syndrome; HF, heart failure; suPAR, soluble urokinase plasminogen activator receptor.

SuPAR at >3.65 ng/mL had 74–83% specificity and 85–96% NPV for all outcomes in overall pre-diagnosed chest pain, confirmed admission-ACS, and convalescent-ACS (see Supplementary material online, *Table S2*). A stepwise rise in HF/death and non-survivors at 5 years was evident with increasing suPAR ranges for all settings, whereas for new HF event, this was evident for acute chest pain and convalescent-ACS patients (*P* < 0.0001) (*Figure 1*).

**Figure 2:**
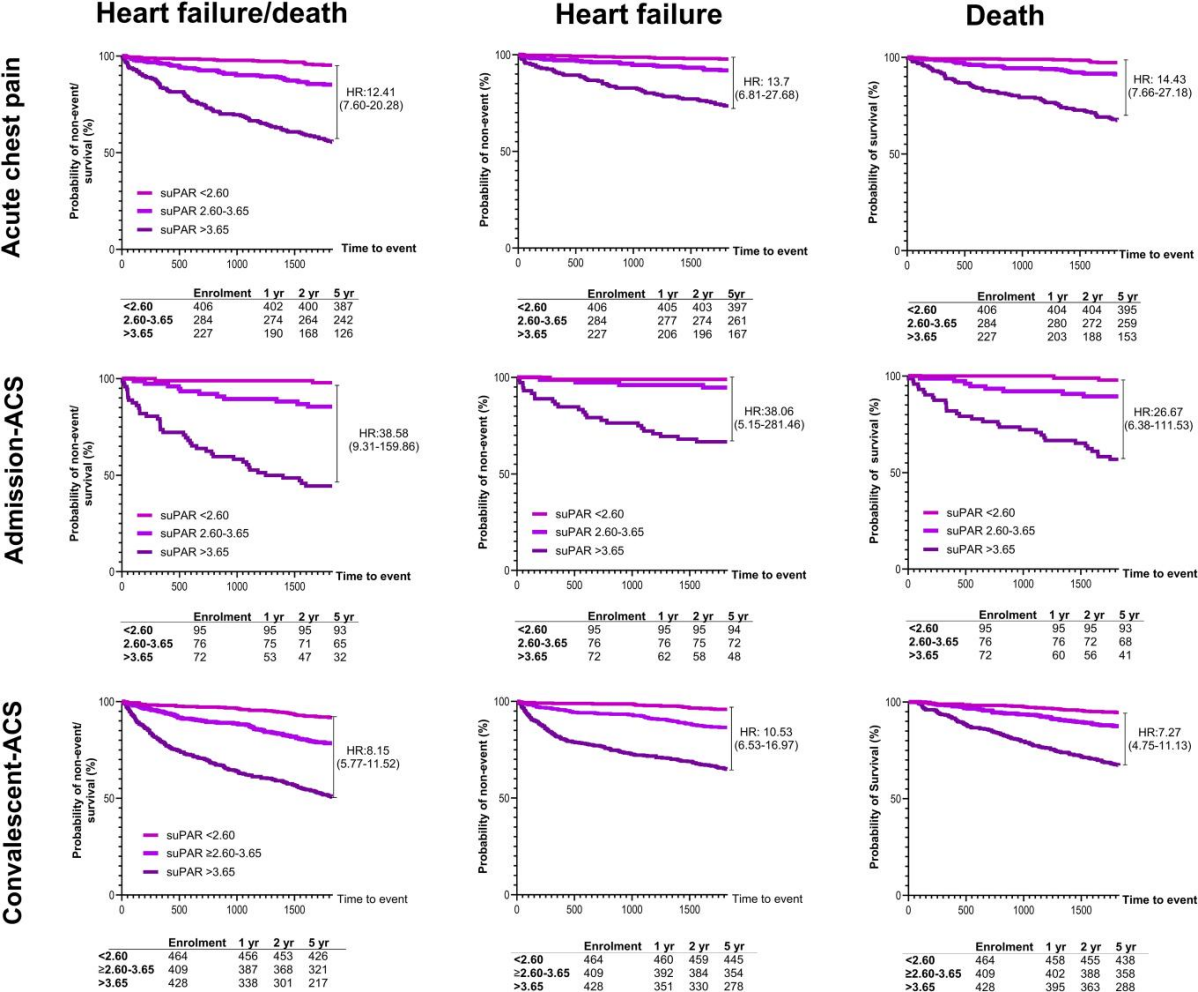
Kaplan–Meier survival curves for suPAR cut-off levels and association with heart failure/death, heart failure, and death in acute chest pain, admission-acute coronary syndrome, and convalescent-acute coronary syndrome. suPAR, soluble urokinase plasminogen activator receptor; HF, heart failure; ACS, acute coronary syndrome.

Unadjusted Cox regression analysis with suPAR < 2.60 ng/mL as the reference indicated suPAR > 3.65 ng/mL in acute chest pain patients increased the hazards of HF/death at 1 year [HR: 14.7 (95% CI: 5.7–37.4), *n* = 54] and at 2 years [HR: 17.2 (7.9–37.7), *n* = 87]. In admission-ACS, suPAR > 3.65 ng/mL demonstrated stronger associations with HF/death at 1 year [HR: 30.7 (4.1–228.7), *n* = 23] and 2 years [HR: 42.6 (5.8–313.9), *n* = 33]. The upper range of suPAR consistently demonstrated strong associations with HF/death at 1 year [HR: 12.2 (6.1–24.1), *n* = 123] and 2 years [HR: 12.5 (7.1–22.2), *n* = 183] in convalescent-ACS. Within 5 years, those in the highest suPAR range were associated with at least >7-fold risks for all outcomes in acute chest pain, in admission-ACS, through to post-ACS recovery (*Figure 2*).

### Prognostic performance of soluble urokinase plasminogen activator receptor, *n*-terminal-pro B-type natriuretic peptide, and troponin I for nominated endpoints

#### Unadjusted analysis

The proportion of patients with clinical endpoints across NT-proBNP and hs-cTnI ranges are shown in Supplementary material online, *Figure S1*. Whilst suPAR and NT-proBNP concentrations were higher for those incurring the endpoints vs. survivors without events in all settings (*P* < 0.0001) (see Supplementary material online, *Table S3*), in admission-ACS higher hs-cTnI concentrations were only obtained in those with a later risk of HF at 5 years (*P* = 0.02).

For risk discrimination, the c-statistic for suPAR was at least ≥0.81 in acute chest pain and ≥0.84 in admission-ACS, compared to ≥0.75 for convalescent-ACS for all endpoints. The c-statistics for individual suPAR and NT-proBNP were higher than the c-statistic for hs-cTnI for all endpoints in all settings (*Table 2*). In admission-ACS, suPAR yielded the highest c-statistic of 0.88 (95% CI: 0.82–0.93) for HF/death, which was better than post-ACS convalescence suPAR of 0.77 (0.74–0.80). Soluble urokinase plasminogen activator receptor combined with NT-proBNP improved risk discrimination, more than the combination of NT-proBNP and hs-cTnI. Combined suPAR and NT-proBNP [0.87 (0.84–0.90)] improved risk discrimination of HF/death instead of suPAR alone [change (Δ) of 0.05, *P* < 0.0001] or NT-proBNP alone (>Δ0.02, *P* = 0.001) in the overall acute chest pain and convalescent-ACS settings. However, as suPAR was the superior biomarker for outcomes in admission-ACS, the combined marker approach did not further augment risk discrimination than using suPAR alone. The c-statistic of hs-cTnI for all endpoints was also improved with the inclusion of suPAR; however, this was not better than using suPAR alone.

**Table 2:** Areas under the curve (c-statistic) for each biomarker for the prediction of outcomes at 5 years for all patients in the acute chest pain, admission-acute coronary syndrome subcohort and convalescent post-acute coronary syndrome cohort

	Acute chest pain cohort (*n* = 917)			Admission-ACS cohort (*n* = 243)			Convalescent-ACS cohort (*n* = 1301)		
	HF/death (*n* = 162)	HF (*n* = 92)	Death (*n* = 110)	HF/death (*n* = 53)	HF (*n* = 29)	Death (*n* = 41)	HF/death (*n* = 337)	HF (*n* = 224)	Death (*n* = 217)
**Biomarker**									
suPAR	0.82 (0.78–0.85)^a^	0.81 (0.76–0.85)^a^	0.82 (0.78–0.86)^a^	0.88 (0.82–0.93)^a^	0.87 (0.81–0.93)^a^	0.84 (0.78–0.90)^a^	0.77 (0.74–0.80)^a^	0.76 (0.73–0.80)^a^	0.75 (0.72–0.79)^a^
hs-cTnI	0.69 (0.65–0.73)	0.71 (0.66–0.75)	0.70 (0.66–0.75)	0.55 (0.48–0.63)	0.62 (0.52–0.71)	0.53 (0.45–0.61)	0.67 (0.64–0.70)	0.68 (0.64–0.71)	0.67 (0.64–0.71)
NT-proBNP	0.85 (0.82–0.88)^a^	0.84 (0.81–0.88)^a^	0.86 (0.82–0.89)^a^	0.83 (0.77–0.89)^a^	0.86 (0.80–0.92)^a^	0.82 (0.75 to 0.90)^a^	0.79 (0.76–0.82)^a^	0.79 (0.76–0.82)^a^	0.77 (0.74–0.80)^a^
**Biomarker combinations**									
suPAR + NT-proBNP	0.87 (0.84–0.90)^b^	0.87 (0.83–0.90)^b^	0.88 (0.84–0.91)^b^	0.89 (0.84–0.94)^c^	0.91 (0.86–0.95)^c^	0.86 (0.80–0.93)^c^	0.82 (0.80–0.85)^b^	0.82 (0.79–0.85)^b^	0.80 (0.77–0.83)^b^
suPAR + hs-cTnI	0.82 (0.78–0.85)	0.81 (0.77–0.86)	0.82 (0.78–0.87)	0.87 (0.81–0.91)	0.88 (0.82–0.93)	0.83 (0.76–0.89)	0.78 (0.75–0.81)	0.78 (0.75–0.81)	0.77 (0.73–0.80)
NT-proBNP + hs-cTnI	0.85 (0.82–0.88)	0.84 (0.80–0.88)	0.86 (0.82–0.89)	0.83 (0.77–0.89)	0.86 (0.80–0.92)	0.83 (0.75–0.90)	0.79 (0.76–0.82)	0.79 (0.76–0.82)	0.77 (0.74–0.80)

^a^Indicates that both soluble urokinase plasminogen activator receptor and *n*-terminal-pro B-type natriuretic peptide were higher than high-sensitivity-cardiac troponin I.

^b^Combined soluble urokinase plasminogen activator receptor and *n*-terminal-pro B-type natriuretic peptide obtained the highest c-statistic.

^c^Combined soluble urokinase plasminogen activator receptor and *n*-terminal-pro B-type natriuretic peptide was higher than combined *n*-terminal-pro B-type natriuretic peptide and high-sensitivity-cardiac troponin I.

suPAR, soluble urokinase plasminogen activator receptor; hs-cTnI; high-sensitivity-cardiac troponin I; NT-proBNP, *n*-terminal-pro B-type natriuretic peptide.

#### Multivariable analysis

**Figure 3:**
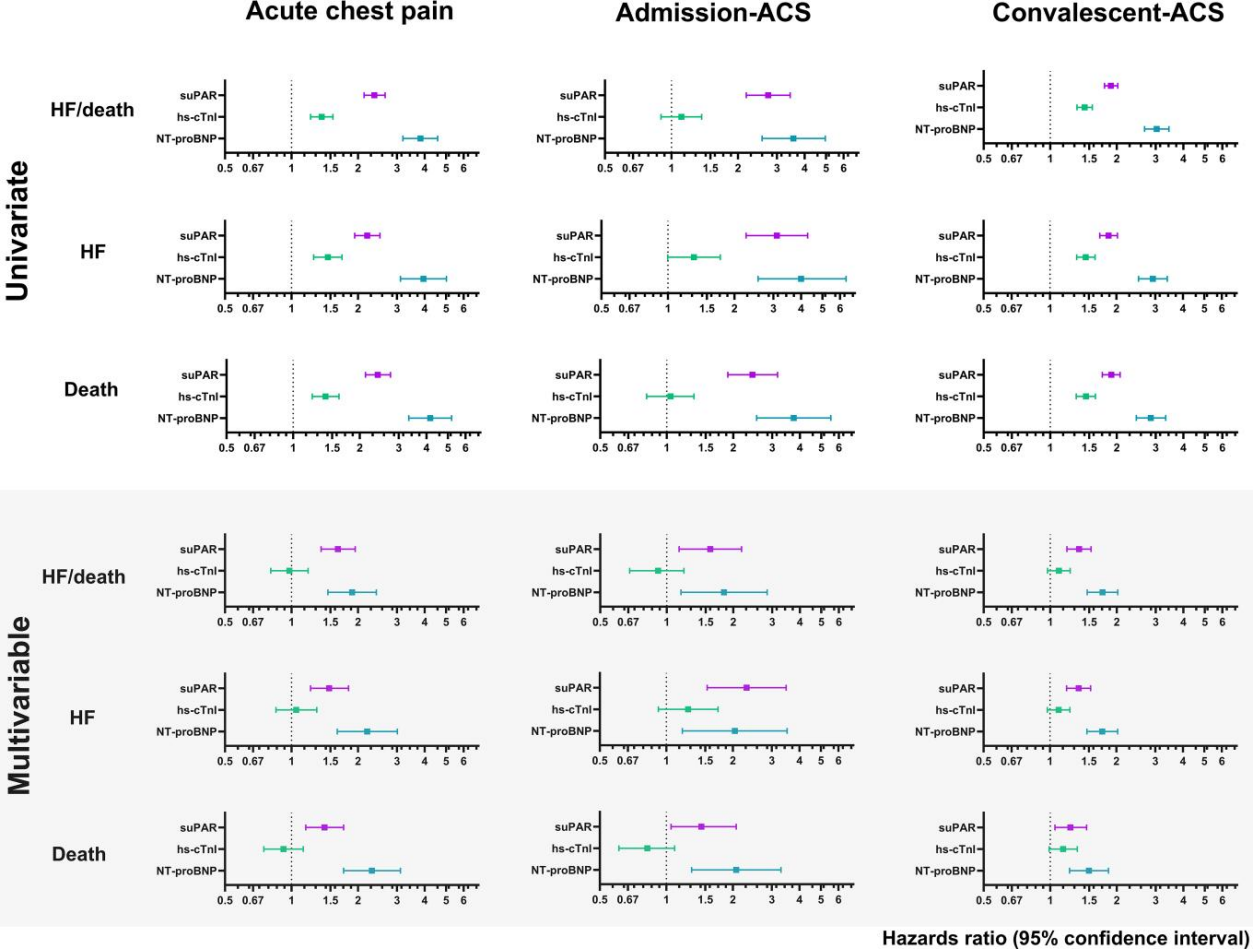
Forrest plot depicting hazard ratios (95% confidence interval) (log_2_ scale) associated with per doubling of soluble urokinase plasminogen activator receptor, high-sensitivity-cardiac troponin I, and *n*-terminal-pro B-type natriuretic peptide concentrations in univariate analyses (*A*) and fully adjusted models (*B*) for heart failure/death, heart failure, and death in overall acute chest pain and the subcohort with admission acute coronary syndrome, and separately for convalescent-acute coronary syndrome. Total event numbers are as follows: heart failure/death (*n* = 162), heart failure (*n* = 92), and death (*n* = 110) in acute chest pain (*n* = 917); heart failure/death (*n* = 53), heart failure (*n* = 29), and death (*n* = 41) in admission-acute coronary syndrome (*n* = 243); heart failure/death (*n* = 337), heart failure (*n* = 224), and death (*n* = 217) in convalescent-acute coronary syndrome (*n* = 1301). suPAR, soluble urokinase plasminogen activator receptor; cTnI, cardiac troponin I; NT-proBNP, n-terminal-pro B-type natriuretic peptide; HF, heart failure; ACS, acute coronary syndrome

Per doubling of unadjusted suPAR in all three settings was strongly associated with obtaining the composite outcome of HF/death with an increased HR ranging from 1.88 (1.76–2.02) to 2.74 (2.17–3.44), *P* < 0.0001 (*Figure 3*). Heart failure/death prediction using suPAR in both acute chest pain and admission-ACS remained independent with HR of >1.57 (1.14–2.18) after adjustment with the inclusion of hs-cTnI and NT-proBNP. Similarly, in convalescent-ACS, suPAR retained independent associations with the composite endpoint [HR: 1.35 (1.19–1.53)]. Continuous suPAR concentrations also independently predicted HF (adjusted HR ≥ 1.35 (1.19–1.54) and death [adjusted HR ≥ 1.23 (1.10–1.50)] across all settings. Associations remained consistent after accounting for the competing risk of all-cause death per doubling of suPAR levels for HF in acute chest pain [aHR: 2.27 (1.52–3.39)] and post-ACS [aHR: 1.84 (1.38–2.47)]. Whilst NT-proBNP remained associated with all clinical endpoints in univariate and multivariable analysis, hs-cTnI was not an independent predictor of the nominated outcomes after adjustment (*Figure 3*).

Soluble urokinase plasminogen activator receptor above the threshold of 3.65 ng/mL during admission in acute chest pain and at 4–6 weeks in convalescent-ACS was associated with adjusted HRs ≥ 2.0 for HF/death (*Table 3*). This relationship was stronger in admission-ACS, with suPAR > 3.65 ng/mL obtaining an adjusted HR of 10.5 (2.4–46.5) for HF/death. In all acute undifferentiated chest pain patients and those with confirmed admission-ACS, suPAR > 3.65 ng/mL also predicted HF readmission and separately for all-cause death, with adjusted HRs of ≥2.2. In convalescent-ACS, those in this highest suPAR cut-off were also associated with an adjusted HR of at least ≥2.0 for HF/death and HF at 5 years, but not with death at 5 years. The highest chosen range for NT-proBNP was independently predictive of all nominated endpoints for all chest pain admission and convalescent-ACS, but there was poor evidence for an association for HF/death prediction in the acute setting of admission-ACS.

**Table 3:** Hazard ratios and 95% confidence interval for soluble urokinase plasminogen activator receptor, ***n*-terminal-pro B-type natriuretic peptide, and high-sensitivity-cardiac troponin I by predetermined cut-off levels for predicting composite heart failure/death, heart failure and death at 5 years**.

	Acute chest pain			Admission-ACS			Convalescent-ACS		
	HF/death (*n* = 162/917)			HF/death (*n* = 53/243)^a^			HF/death (*n* = 337/1301)		
*Model*	suPAR	hs-cTnI	NT-proBNP	suPAR	hs-cTnI	NT-proBNP	suPAR	hs-cTnI	NT-proBNP
*(A) Univariate*									
C1: Ref	1	1	1	1	1	1	1	1	1
>C1–C2	3.5 (1.9–5.8)	4.0 (2.6–6.0)	4.4 (2.8–7.0)	7.3 (1.6–32.9)	9.9 (1.3–77.4)	1.5 (0.5–4.6)	2.9 (1.9–4.1)	2.8 (2.0–4.1)	3.2 (2.0–5.0)
>C2	12.4 (7.6–20.3)	4.0 (2.7–6.0)	12.5 (8.7–17.9)	38.6 (9.3–159.9)	10.4 (1.4–75.3)	9.2 (4.9–17.4)	8.2 (5.8–11.5)	4.7 (3.3–6.8)	9.0 (6.1–13.3)
*(B) Multivariable*									
C1: Ref	1	1	1	1	1	1	1	1	1
>C1–C2	1.6 (0.9–2.9)	1.0 (0.6–1.6)	1.5 (0.9–2.5)	5.4 (1.2–24.6)	3.1 (0.4–25.4)	0.8 (0.3–2.6)	1.4 (1.0–2.1)	1.5 (1.1–2.3)	1.7 (1.1–2.7)
>C2	2.4 (1.3–4.4)	1.1 (0.7–1.8)	2.7 (1.7–4.3)	10.5 (2.4–46.5)	2.8 (0.4–21.1)	1.8 (0.9–3.8)	2.0 (1.3–2.8)	1.9 (1.3–2.9)	2.7 (1.8–4.2)

	HF (*n* = 92/917)^a^			HF (*n* = 29/243)^a,b^			HF (*n* = 224/1301)			
*(A) Univariate*									
C1: Ref	1	1	1	1	1	1	1	1	1
>C1–C2	3.8 (1.7–8.1)	5.1 (2.8–9.1)	3.8 (2.0–7.4)				3.4 (2.0–5.8)	3.4 (2.1–5.6)	4.5 (2.4–8.5)
>C2	13.7 (6.8–27.7)	5.5 (3.1–9.6)	12.5 (7.8–20.3)	10.9 (4.4–26.8)	3.2 (1.1–9.1)	13.3 (5.4–32.7)	10.5 (6.5–17.0)	6.2 (3.8–10.1)	12.8 (7.3–22.5)
*(B) Multivariable*									
C1: Ref	1	1	1	1	1	1	1	1	1
>C1–C2	3.0 (1.4–6.5)	2.3 (1.2–4.1)	2.5 (1.3–4.9)				1.8 (1.0–3.1)	1.7 (1.0–2.9)	2.3 (1.2–4.4)
>C2	4.7 (2.2–10.3)	2.0 (1.0–3.9)	4.1 (2.2–7.5)	3.9 (1.4–11.2)	2.6 (0.9–7.5)	4.5 (1.6–12.8)	2.5 (1.5–4.2)	2.3 (1.3–3.8)	3.6 (1.9–6.6)

	Death (*n* = 110/917)^a^			Death (*n* = 41/243)^a,b^			Death (*n* = 217/1301)		
*(A) Univariate*									
C1: Ref	1	1	1	1	1	1	1	1	1
>C1–C2	3.4 (1.7–6.8)	3.9 (2.3–6.5)	5.6 (3.0–10.2)				2.4 (1.5–3.8)	3.0 (1.8–4.7)	2.7 (1.5–4.7)
>C2	14.4 (7.7–27.2)	4.7 (2.9–7.6)	16.9 (10.5–27.1)	8.3 (4.2–16.6)	1.8 (0.9–3.8)	8.5 (4.3–16.7)	7.3 (4.8–11.1)	4.8 (3.0–7.5)	8.2 (5.1–13.2)
*(B) Multivariable*									
C1: Ref	1	1	1	1	1	1	1	1	1
>C1–C2	2.2 (1.1–4.5)	1.3 (0.8–2.3)	2.9 (1.6–5.6)				1.1 (0.6–1.7)	1.5 (0.9–2.4)	1.2 (0.7–2.1)
>C2	3.5 (1.7–7.2)	1.2 (0.7–2.1)	4.7 (2.6–8.4)	2.2 (1.0–5.1)	1.0 (0.5–2.1)	2.3 (1.1–5.1)	1.4 (0.9–2.1)	1.7 (1.0–2.9)	1.9 (1.2–3.2)

Results are presented for the acute chest pain cohort including the admission-acute coronary syndrome subcohort and the convalescent-acute coronary syndrome cohort

Soluble urokinase plasminogen activator receptor, high-sensitivity cardiac troponin I, and *n*-terminal-pro B-type natriuretic peptide according to cut-off values in univariate (A) and multivariable adjustment (B). Multivariable modelling included all three biomarkers cut-off levels compared against cut-off 1 (C1) as the reference, adjusting for age, sex, history of hypertension, history of heart failure, history of myocardial infarction, heart rate, systolic blood pressure, diabetes, DAPT, dual antiplatelet therapy, creatinine, and in-hospital PCI. Cut-off ranges are as follows: soluble urokinase plasminogen activator receptor, C1 < 2.60 ng/mL, C1–C2 ≥ 2.60–3.65 ng/mL, and C2 > 3.65 ng/mL; high-sensitivity cardiac troponin I, CI < 5.0 ng/L, C1–C2 ≥ 5.0–14.9 ng/L, and C3 ≥ 15.0 ng/L; and *n*-terminal-pro B-type natriuretic peptide, C1 < 49.9 pmol/L, C1–C2 ≥ 50.0–99.9 pmol/L, and C3 ≥ 100.0 pmol/L.

ACS, acute coronary syndrome; HF, heart failure; soluble urokinase plasminogen activator receptor, suPAR; hs-cTnI, high-sensitivity cardiac troponin; NT-proBNP, *n*-terminal-pro B-type natriuretic peptide.

^a^For the acute chest pain cohort and admission-ACS subcohort, where *n* < 130, adjustment included a probability score derived from logistic regression combining all the abovementioned adjustment variables and biomarkers were added to the model accordingly.

^b^Due to the limited numbers for events in admission-ACS, cut-off ranges were compared between suPAR > 3.65 ng/mL vs. ≤3.65 ng/mL, hs-cTnI > 15 ng/L vs. ≤15 ng/L and NT-proBNP > 100 pmol/L vs. ≤100 pmol/L.

Soluble urokinase plasminogen activator receptor at the upper range remained strongly associated with a higher risk of obtaining HF/death within 5 years in both women and men in acute chest pain and convalescent-ACS. However, the small number of women in admission-ACS with HF/death leads to wide confidence intervals with less certainty of any independent associations with this endpoint at each biomarker’s respective upper concentrations (see Supplementary material online, *Table S4*). Men classified into the upper suPAR range in admission-ACS demonstrated strong associations with the composite outcome obtaining an adjusted HR of 3.6 (1.4–9.0), whilst evidence for NT-proBNP and hs-cTnI having increased hazard for the composite outcome was poor. In renal insufficiency, upper levels of suPAR remained strongly associated with risks of HF/death in all three settings.

## Discussion

This study reports on the predictive performance of plasma suPAR concentrations measured at key points in the clinical patient journey from acute, as yet undiagnosed chest pain, in confirmed admission-ACS, through to post-ACS convalescence. Soluble urokinase plasminogen activator receptor powerfully predicts future adverse cardiovascular outcomes in patients during long-term follow-up, complementing established cardiovascular biomarkers. Our study leverages the prospectively planned acquisition of admission samples from an undifferentiated chest pain cohort of heterogeneous aetiology including a substantial subgroup with subsequently confirmed ACS and the inclusion of independently collected convalescent samples from a post-ACS cohort. SuPAR was assessed alongside matched hs-cTnI and NT-proBNP in well-annotated ‘real-life’ cohorts with detailed clinical information and long-term follow-up covering the full range of presentations with and without coronary involvement. The predictive performance of a suPAR threshold derived from the upper limit of a healthy volunteer cohort was demonstrated across the clinical ACS spectrum, showing an independent association with a higher risk of subsequent risk of combined HF/death or HF readmission.

Despite the essential roles of cardiac troponins and NT-proBNP in the accurate diagnosis of ACS and HF, respectively, their prognostic value may be more limited. We demonstrated that the predictive power of suPAR for nominated endpoints was comparable to NT-proBNP but superior to hs-cTnI. As shown, the combined or multi-marker approach for risk stratification may be advantageous, considering that multiple mechanistic pathways are associated with adverse cardiac outcomes. Unlike the two cardiac markers, suPAR potentially reflects the chronic inflammatory contribution to adverse ventricular remodelling and the ongoing atheromatous process. Thus, the addition of suPAR to NT-proBNP augmented risk discrimination for all endpoints across all assessed settings, which was superior to the combined approach of NT-proBNP and hs-cTnI.

Interestingly, suPAR consistently predicted HF readmission within 5 years of the index hospital presentation in all three defined groups. However, the biological relationship between suPAR and HF is unclear. Our results show strong associations between suPAR and NT-proBNP across the three settings, which is a relationship that is also present in healthy subjects^21^ and patients with HF.^22,23^ Intriguingly, the underlying connections between the myocardium, suPAR, and NT-proBNP appear to be in opposing directions. We previously documented suPAR concentrations to be lower in the coronary sinus, indicating extraction of suPAR across the heart,^21^ in contradistinction to the known net production of NT-proBNP by the heart. In the current context, inflammation, a key element contributing to ACS events and during progression to adverse remodelling and overt clinical HF, could drive production of suPAR and NT-proBNP. However, this leaves the question as to whether suPAR and NT-proBNP influence one another directly or respond in parallel but independently when exposed to one or more pathological stimuli. Future studies should therefore examine whether suPAR is mechanistically causative of cardiovascular disease progression or may counteract disease processes and whether management guided by suPAR levels may help prevent HF development.

In the acute setting, admission suPAR concentrations appear to confer greater risks of adverse outcomes in admission-ACS than convalescent post-ACS levels despite similar median values between the two ACS cohorts. However, the intentional measurement of suPAR in convalescence avoiding the volatile biomarker fluctuations often present during acute presentations confirms that high suPAR concentrations in the recovery phase are still independently associated with adverse outcomes. Our results agree with others showing suPAR concentrations to be higher in women than men.^21,24^ Although higher cut-offs may be more appropriate for women,^24^ our current study was not adequately powered to examine accurate cut-offs for women and men for risk prediction. Instead, our results showing suPAR at >3.65 ng/mL being consistently predictive of HF/death in both sexes in the overall acute chest pain setting and in convalescent-ACS suggests that this level might trigger closer surveillance and more aggressive up-titration of pharmacotherapy in treatment strategies for chest pain/ACS. It is however unclear whether treatment guidance using suPAR is beneficial due to limited literature surrounding this topic. In the setting of COVID-19, suPAR concentrations have been used in stratifying patients to receive anakinra, which subsequently improved outcomes.^25^ Precision CAD, an ongoing randomized clinical trial, may provide a better evaluation of suPAR in the current context, to complement cardiac biomarkers in guiding CAD risk reduction (NCT04755413). Future research should also extend on evaluating whether direct modulation of suPAR is a possible pathway to ameliorate cardiovascular disease progression.

Limitations are associated with the small number of events in admission-ACS that hindered full adjustment in multivariable analyses, the inability to include other novel inflammatory markers for comparison, concentrations assessed from biobanked samples, and also the first analysis of a convalescence cohort without data in the acute phase. Patients with missing clinical variables for multivariable analyses from the overall study population were also excluded; however, we expect any effects from this to be negligible, considering that prognostic assessments of all biomarkers and risk factors were conducted in the same manner. Strengths of our study include leveraging long-term follow-up, large multi-centre sample sizes, adherence to the Strengthening the Reporting of Observational studies in Epidemiology guidelines, inclusion of the full spectrum of undifferentiated chest pain and ACS presentations to include both acute and recovery settings, associated comorbidities, and generalizability of ‘real-world’ risk analysis.

In summary, we have detailed the independent utility of suPAR as an indicator of long-term prognosis, generalized application in three settings, following the potential journey of a patient from hospital admission through to the recovery convalescent phase of ACS. Plasma suPAR, measured either at hospital admission in acute chest pain and admission-ACS or at 4–6 weeks following recovery from ACS, strongly predicted risk for obtaining the composite outcome of HF/death, independent of key clinical indicators and cardiac markers at all points of the patient journey.

## Lead author biography


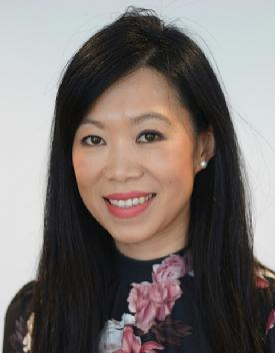


Dr Janice Chew-Harris is a research fellow at the Department of Medicine, University of Otago, Christchurch, New Zealand. She has a special interest in novel biomarkers and their clinical diagnostic or prognostic applications in acute coronary syndromes and heart failure. She also has a keen interest in preclinical models of myocardial infarction and assessing the therapeutic potential of proteins.

## Supplementary Material

oeaf097_Supplementary_Data

## Data Availability

The data underlying this article can be shared on reasonable request to the corresponding author.
