# Supplementary material for: Soluble urokinase plasminogen activator receptor for risk stratification from undifferentiated acute chest pain through to convalescence after acute coronary syndromes
Source: Eur Heart J Open. 2025 Aug 7;5(5):oeaf097. doi: 10.1093/ehjopen/oeaf097 (PMC13158661; doi:10.1093/ehjopen/oeaf097)
Supplement: oeaf097_Supplementary_Data [file oeaf097_supplementary_data.docx]

| **Supplemental Table 1:** Independent associates of plasma suPAR measured at admission for acute chest pain patients and acute-ACS and separately at 1-month convalescence in post-ACS | | | | | | | | | |
| --- | --- | --- | --- | --- | --- | --- | --- | --- | --- |
|  | **Acute chest pain (n=917)** | | | **Acute-ACS (n=243)** | | | **Convalescence-ACS (n=1301)** | | |
|  | Mean Square | F | P-value | Mean Square | F | P-value | Mean Square | F | P-value |
| Age | 97.98 | 46.716 | <0.0001 | 35.44 | 21.532 | <0.0001 | 80.997 | 29.525 | <0.0001 |
| Sex | 30.386 | 14.488 | 0.008 | 7.075 | 4.299 | 0.04 | 40.611 | 14.804 | <0.0001 |
| SBP | 10.452 | 4.983 | 0.03 | 3.288 | 1.997 | 0.16 | 2.02 | 0.737 | 0.39 |
| DBP | 12.221 | 5.827 | 0.02 | 0.288 | 0.175 | 0.68 | 7.504 | 2.735 | 0.098 |
| HR | 14.427 | 6.879 | 0.009 | 0.063 | 0.038 | 0.85 | 13.274 | 4.839 | 0.03 |
| Hx- Hypertension | 2.898 | 1.382 | 0.24 | 0.981 | 0.596 | 0.44 | 3.329 | 1.214 | 0.27 |
| Hx-MI | 10.049 | 4.791 | 0.03 | 6.797 | 4.13 | 0.04 | 0.194 | 0.071 | 0.79 |
| DM | 0.013 | 0.006 | 0.94 | 0.009 | 0.006 | 0.94 | 4.489 | 1.636 | 0.20 |
| Hx-HF | 1.774 | 0.846 | 0.36 | 4.963 | 3.061 | 0.08 | 14.541 | 5.300 | 0.02 |
| Creatinine | 266.03 | 126.84 | <0.0001 | 37.754 | 22.939 | <0.0001 | 283.17 | 103.224 | <0.0001 |
| Second anti-platelet therapy | 0.009 | 0.004 | 0.95 | 0.087 | 0.053 | 0.82 | 0.261 | 0.095 | 0.76 |
| In-hospital PCI | 2.358 | 1.179 | 0.28 | 2.008 | 1.220 | 0.27 | 3.526 | 1.285 | 0.26 |
| NT-proBNP | 49.97 | 23.83 | <0.0001 | 0.091 | 0.055 | 0.81 | 82.60 | 30.11 | <0.0001 |
| Hs-cTnI | 3.608 | 1.72 | 0.19 | 8.07 | 4.904 | 0.03 | 0.01 | 0.00 | 0.99 |
| Adjusted R^2^ | 0.329 | | | 0.325 | | | 0.244 | | |

Note: All variables used in the analysis correspond to the timing of suPAR sampling.

| **Supplemental Table 2** Sensitivity and specificity of plasma suPAR using predetermined cut-off of 3.65 ng/mL. Results in brackets are 95% confidence intervals | | | |
| --- | --- | --- | --- |
|  | **HF/death** | **HF** | **Death** |
| **Acute Chest Pain (n=917)** | | | |
| **suPAR >3.65 ng/mL**  Sensitivity  Specificity  NPV PPV | 61.7 (54.1-68.9)  83.4 (80.6-85.9)  91.0 (88.7-92.9)  44.4 (38.1-51.0) | 64.1 (54.2-80.7)  79.9 (77.0-82.5)  95.2 (93.4-96.6)  26.2 (20.9-32.3) | 66.4 (57.1-74.5)  81.2 (78.3-83.7)  94.7 (92.7-96.1)  32.4 (26.7-38.8) |
| **Admission-ACS (n=243)** | | | |
| **suPAR >3.65 ng/mL**  Sensitivity  Specificity  NPV PPV | 73.6 (60.4-83.6)  83.2 (77.2-95.1)  91.9 (86.8-95.1)  54.9 (43.4-66.0) | 79.3 (61.6-90.2)  77.6 (71.5-82.6)  96.5 (92.6-98.4)  32.4 (22.7-43.9) | 73.2 (58.1-84.3)  79.7 (73.6-84.7)  93.6 (88.9-96.4)  42.3 (31.5-53.8) |
| **Convalescent-ACS (n=1301)** | | | |
| **suPAR >3.65**  Sensitivity  Specificity  NPV PPV | 62.6 (57.3-67.6)  77.5 (74.7-80.0)  85.6 (83.1-87.7)  49.3 (44.6-54.0) | 67.0 (60.6-72.8)  74.2 (71.5-76.7)  91.5 (89.5-93.2)  35.0 (30.7-39.7) | 65.0 (58.4-71.0)  73.5 (70.8-76.0)  91.3 (89.2-93.0)  32.9 (28.7-37.5) |

**
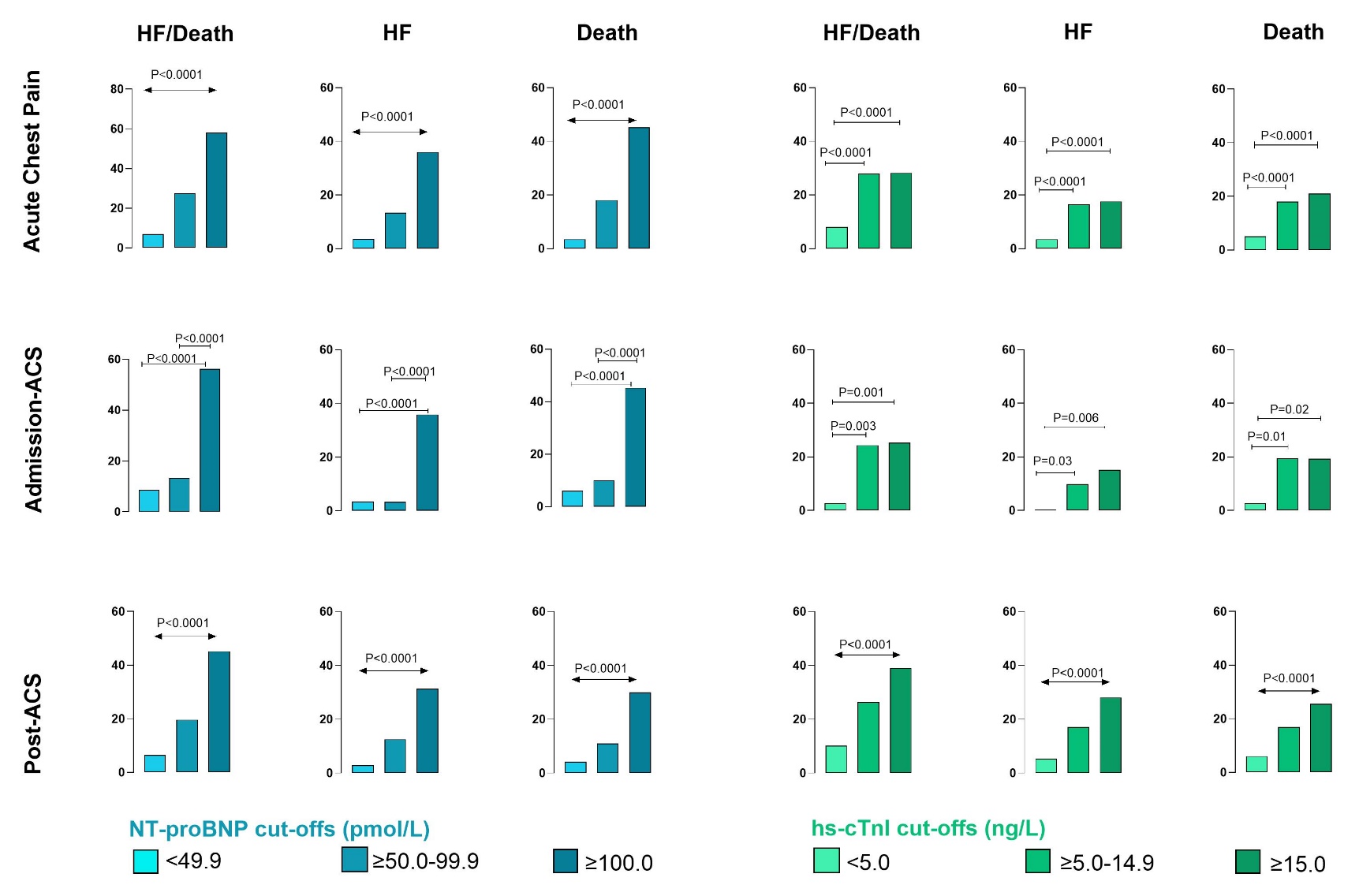
**

**Supplemental Figure 1:** Bar graphs depict the proportion of patients incurring outcomes at 5 years according to NT-proBNP cut-off (blue) and hs-cTnI (green) levels. Unless otherwise stated, the double-headed arrow denotes differences across all levels, i.e. increasing rates of all outcomes for those in the upper-level vs mid-level, upper-level vs lowest level, and separately the mid-level vs lowest level.

| **Supplemental Table 3** Biomarker concentrations for patients with events and those who did not reach the primary endpoint of outcomes at 5 years post-index admission.  Data are presented as median and inter-quartile range for acute chest pain, admission ACS and separately for convalescent-ACS patients | | | | | | | | | | | |
| --- | --- | --- | --- | --- | --- | --- | --- | --- | --- | --- | --- |
| **Biomarker** | **Acute chest pain cohort** | | | **Admission-ACS cohort** | | | | **Convalescent-ACS cohort** | | | |
|  | **Event** | **Non-events** | **P value** | **Event** | **Non-events** | | **P value** | **Event** | | **Non-events** | **P value** |
|  | HF/death  (n=162) | Survival without HF  (n=755) |  | HF/death  (n=53) | Survival without HF (n=190) | |  | HF/death  (n=337) | | Survival without HF (n=964) |  |
| suPAR (ng/mL) | 4.2 (3.3-5.4) | 2.6 (2.1-3.3) | <0.0001 | 4.5 (3.6-5.7) | 2.7 (2.2-3.3) | | <0.0001 | 4.0 (3.2-5.5) | | 2.8 (2.3-3.6) | <0.0001 |
| hs-cTnI (ng/L) | 12.0 (5.0-29.0) | 3.9 (2.0-12.0) | <0.0001 | 51.2 (16.5-155.7) | 44.0 (7.0-181.1) | | 0.25 | 14.3 (7.6-31.9) | | 7.6 (4.1-15.7) | <0.0001 |
| NT-proBNP (pmol/L) | 115 (43-236) | 16 (6-39) | <0.0001 | 140 (54-328) | 26 (11-61) | | <0.0001 | 170 (94-303) | | 60 (29-118) | <0.0001 |
|  | HF readmission  (n=92) | No HF readmission  (n=825) |  | HF readmission  (n=29) | No HF readmission  (n=214) | |  | HF readmission  (n=224) | | No HF readmission  (n=1077) |  |
| suPAR (ng/mL) | 4.3 (3.3-5.3) | 2.7 (2.1-3.5) | <0.0001 | 4.8 (3.8-6.3) | 2.7 (2.2-3.6) | | <0.0001 | 4.2 (3.3-5.6) | | 2.9 (2.3-3.7) | <0.0001 |
| hs-cTnI (ng/L) | 12.0 (6.0-33.8) | 4.0 (2.0-12.5) | <0.0001 | 75.3 (22.6-227.4) | 42.1 (9.1-161.9) | | 0.04 | 16.0 (8.1-33.3) | | 7.9 (4.2-16.6) | <0.0001 |
| NT-proBNP (pmol/L) | 136 (49-269) | 18 (6-45) | <0.0001 | 199 (103-360) | 29 (11-72) | | <0.0001 | 182 (106-327) | | 66 (32-132) | <0.0001 |
|  | All-cause death  (n=110) | Survival  (n=807) |  | All-cause death (n=41) | | Survival  (n=202) |  | All-cause death (n=217) | Survival  (n=1084) | |  |
| suPAR (ng/mL) | 4.4 (3.4-5.9) | 2.6 (2.1-3.4) | <0.0001 | 4.4 (3.6-5.6) | | 2.7 (2.2-3.4) | <0.0001 | 4.2 (3.3-5.6) | 2.9 (2.3-3.7) | | <0.0001 |
| hs-cTnI (ng/L) | 13.9 (5.8-32.2) | 4.0 (2.0-12.5) | <0.0001 | 51.0 (16.0-127.5) | | 44.5 (8.8-182.7) | 0.54 | 14.7 (8.1-36.0) | 7.9 (4.3-17.7) | | <0.0001 |
| NT-proBNP (pmol/L) | 134 (61-284) | 17 (6-42) | <0.0001 | 199 (61-360) | | 28 (11-67) | <0.0001 | 177 (100-313) | 67 (32-134) | | <0.0001 |

| **Supplemental Table 4** Hazard ratios and 95% confidence intervals for suPAR, NT-proBNP and hs-Troponin I by predetermined cut-off levels for predicting composite heart failure/death at 5 years. Results are presented for the acute chest pain cohort, the admission-ACS sub-cohort and the convalescent-ACS cohort. Total numbers for women and men are presented as events (n) / total patients (n). | | | | | | | | | | | | | | | | | | | | |
| --- | --- | --- | --- | --- | --- | --- | --- | --- | --- | --- | --- | --- | --- | --- | --- | --- | --- | --- | --- | --- |
|  | **Acute chest pain** | | | | **Admission-ACS** | | | | | | | **Convalescent-ACS** | | | | | | |  |  |
|  | suPAR | hs-cTnI | | NT-proBNP | suPAR | | hs-cTnI | | | NT-proBNP | | suPAR | | hs-cTnI | | NT-proBNP | | |  |  |
|  | **Women (n=54/316) *** | | | | **Women (n=16/65) *** | | | | | | | **Women (n=108/360) *** | | | | | | |  |  |
| *(A) Univariate* | | |  |  |  |  | | |  | | |  |  | |  | | |  |  |  |
| C1: Ref | 1 | | 1 | 1 | 1 | 1 | | | 1 | | | 1 | 1 | | 1 | | |  |  |  |
| >C1-C2 | 17.8 (2.4-132.6) | | 5.8 (3.1-10.9) | 5.7 (2.5-12.9) |  |  |  |  |  |  |  | 4.4 (1.9-10.5) | 2.9 (1.7-5.1) | | 3.7 (1.5-9.2) | | |  |  |  |
| >C2 | 46.8 (6.4-341.6) | | 3.8 (1.6-7.0) | 14.9 (7.5-29.8) | 4.5 (1.6-13.1) | 1.1 (0.4-3.0) | | | 9.3 (2.6-32.7) | | | 8.2 (3.5-18.8) | 4.6 (2.6-8.2) | | 8.5 (3.7-19.6) | | |  |  |  |
| *(B) Multivariable* | | | |  |  | | |  | | |  |  | |  | | |  | | |  |
| C1: Ref | 1 | | 1 | 1 | 1 | 1 | | | 1 | | | 1 | 1 | | 1 | | |  |  |  |
| >C1-C2 | 10.6 (1.4-80.0) | | 1.5 (0.7-3.1) | 2.5 (1.0-6.0) |  |  | | |  | | | 2.6 (1.1-6.3) | 1.5 (0.9-2.7) | | 2.2 (0.9-5.5) | | |  |  |  |
| >C2 | 13.9 (1.8-104.8) | | 1.5 (0.7-3.2) | 4.3 (1.9-9.7) | 1.6 (0.5-5.3) | 1.3 (0.4-3.6) | | | 3.6 (0.9-13.9) | | | 2.8 (1.2-6.7) | 2.2 (1.2-4.0) | | 3.3 (1.3-8.0) | | |  |  |  |
|  | **Men (n=108/601) *** | | | | **Men (n=37/178) *** | | | | | | | **Men (n=229/941)** | | | | | | |  |  |
| 1. *Univariate* | | |  |  |  |  | | |  | | |  |  | |  | | |  |  |  |
| C1: Ref | 1 | | 1 | 1 | 1 | 1 | | | 1 | | | 1 | 1 | | 1 | | |  |  |  |
| >C1-C2 | 2.5 (1.3-4.6) | | 3.1 (1.8-5.3) | 4.0 (2.2-7.2) |  |  | | |  | | | 2.4 (1.5-3.7) | 2.9 (1.8-4.7) | | 2.9 (1.8-4.9) | | |  |  |  |
| >C2 | 11.5 (6.8-19.4) | | 4.2 (2.6-6.9) | 11.9 (7.8-18.3) | 13.2 (6.2-28.1) | 3.1 (1.2-7.8) | | | 8.6 (4.4-16.8) | | | 8.6 (5.9-12.6) | 5.2 (3.3-8.3) | | 9.2 (5.9-14.2) | | |  |  |  |
| *(B) Multivariable* | | | | |  | | |  | | |  |  | |  | | |  | | |  |
| C1: Ref | 1 | | 1 | 1 | 1 | 1 | | | 1 | | | 1 | 1 | | 1 | | |  |  |  |
| >C1-C2 | 1.5 (0.8-2.9) | | 1.2 (0.7-2.1) | 1.6 (0.9-3.1) |  |  | | |  | | | 1.1 (0.7-1.8) | 1.4 (0.8-2.3) | | 1.5 (0.9-2.7) | | |  |  |  |
| >C2 | 2.4 (1.2-4.6) | | 1.2 (0.7-2.1) | 2.3 (1.3-4.2) | 3.6 (1.4-9.0) | 1.3 (0.4-3.6) | | | 1.5 (0.7-3.3) | | | 1.8 (1.2-2.7) | 1.7 (1.0-2.9) | | 2.4 (1.4-4.0) | | |  |  |  |
|  | **eGFR < 60 mL/min/1.73m^2^ (n=124/452) *** | | | | **eGFR < 60 mL/min/1.73m^2^ (n=45/146) *** | | | | | | | **eGFR < 60 mL/min/1.73m^2^ (n=151/285)** | | | | | |  |  |  |
| 1. *Univariate* | | | | |  |  | | |  | | |  |  | |  | | |  |  |  |
| C1: Ref | 1 | | 1 | 1 | 1 | 1 | | | 1 | | | 1 | 1 | | 1 | | |  |  |  |
| >C1-C2 | 3.5 (1.6-7.5) | | 2.7 (1.7-4.6) | 2.3 (1.3-4.2) |  |  |  |  |  |  |  | 1.3 (0.5-3.6) | 3.6 (1.7-8.0) | | 1.9 (0.8-4.5) | | |  |  |  |
| >C2 | 11.5 (5.7-22.8) | | 3.3 (2.0-5.3) | 7.8 (5.2-11.8) | 8.0 (3.9-16.2) | 1.6 (0.8-3.2) | | | 7.2 (1.8-28.7) | | | 4.9 (2.0-12.1) | 5.5 (2.6-12.0) | | 4.3 (2.0-9.1) | | |  |  |  |
| *(B) Multivariable* | | | | |  |  | | |  | | |  |  | |  | | |  |  |  |
| C1: Ref | 1 | | 1 | 1 | 1 | 1 | | | 1 | | | 1 | 1 | | 1 | | |  |  |  |
| >C1-C2 | 2.0 (0.9-4.3) | | 1.1 (0.6-1.8) | 0.7 (0.4-1.4) |  |  |  |  |  |  |  | 1.0 (0.4-2.8) | 2.0 (0.9-4.6) | | 1.1 (0.4-2.4) | | |  |  |  |
| >C2 | 3.3 (1.5-6.8) | | 1.2 (0.7-2.0) | 0.9 (0.5-1.8) | 2.9 (1.3-6.3) | 0.9 (0.5-2.0) | | | 1.6 (0.9-3.3) | | | 2.7 (1.1-6.7) | 2.1 (0.9-5.0) | | 1.9 (0.8-4.2) | | |  |  |  |

suPAR, hs-cTnI and NT-proBNP according to cut-off values in univariate (A) and multivariable adjustment (B).

Multivariable modelling included all three biomarker cut-off levels compared against cut-off 1 (C1) as the reference, adjusting for age, history of hypertension, history of HF, history of MI, heart rate, SBP, diabetes, DAPT, creatinine, and in-hospital PCI. Cut-off concentrations are: suPAR: C1: <2.60 ng/mL, C1-C2: ≥2.60-3.65 ng/mL and C2:>3.65 ng/mL, hs-cTnI: CI: <5.0 ng/L, C1-C2: ≥5.0-14.9 ng/L and C3: >15.0 ng/L, NT-proBNP: C1: <49.9 pmol/L, C1-C2: ≥50.0-99.9 pmol/L and C3: ≥100.0 pmol/L.

* For all settings, when event numbers (n) are <130, adjustment included a probability score derived from logistic regression combining all the abovementioned adjustment variables. Biomarkers were added to the model accordingly, and comparisons entailed individual biomarkers plus the probability score containing the independent biomarkers. Due to the limited numbers for events according to sex divisions or eGFR in the acute-ACS sub-cohort, cut-off values were compared between suPAR >3.65 ng/mL vs ≤3.65 ng/mL, hs-cTnI >15 ng/L vs ≤15 ng/L and NT-proBNP >100 pmol/L vs ≤100 pmol/L. eGFR was calculated using the CKD-EPI formula for acute chest pain patients including the subcohort diagnosed with ACS, and the MDRD formula was used for the post-ACS cohort
